# Case Report: A myxoma with a far reach

**DOI:** 10.3389/fcvm.2024.1340406

**Published:** 2024-01-24

**Authors:** Elias Akiki, Arman Arghami, Muhannad A. Abbasi, Edward A. El-Am, Ali Ahmad, Thomas A. Foley, Richard C. Daly, Joseph J. Maleszewski, Reto Kurmann, Kyle W. Klarich

**Affiliations:** Department of Cardiovascular Medicine, Mayo Clinic, Rochester, MN, United States

**Keywords:** atypical myxoma, multimodal imaging, 3D reconstruction, pulmonary veins, cardiac MRI (CMRI), cardiac CT, echocardiography

## Abstract

A 73-year-old woman presented to the emergency department with a syncopal episode and a history of dizzy spells. A transthoracic echocardiogram demonstrated a large left atrial mass extending into the right upper pulmonary veins. Subsequently, cardiac magnetic resonance imaging and coronary computed tomography angiography with three-dimensional reconstruction and printing of the heart and mass were performed, which demonstrated a high index of suspicion for an atypical left atrial myxoma. The mass was excised robotically, and the pathology report confirmed a diagnosis of myxoma.

## Introduction

Cardiac myxoma is one of the most common primary cardiac neoplasms in adults, second only to papillary fibroelastomas (1). Patients with cardiac myxomas can present with a variety of symptoms such as chest pain, shortness of breath, palpitations, and embolic phenomena. Malaise or syncope was reported in 14% of patients in a case series describing 112 cases of left atrial myxoma (2).

## Case report

### History of presentation

A 73-year-old woman presented to an outside clinic complaining of dizzy spells leading to a single syncopal episode. Following the syncopal episode, she was taken to the emergency department (ED). She was noted to have supraventricular tachycardia (SVT). An echocardiogram demonstrated a left atrial mass. However, she did not report any symptoms such as chest pain, dyspnea, orthopnea, paroxysmal nocturnal dyspnea, palpitations, or irregular heart rhythm. She was referred to our institution for further evaluation and treatment of the mass.

### Past medical history

The patient had a history of type 2 diabetes mellitus (on oral agents), well-controlled systemic hypertension, hyperlipidemia, and bronchial asthma.

### Investigations

**Figure 1:**
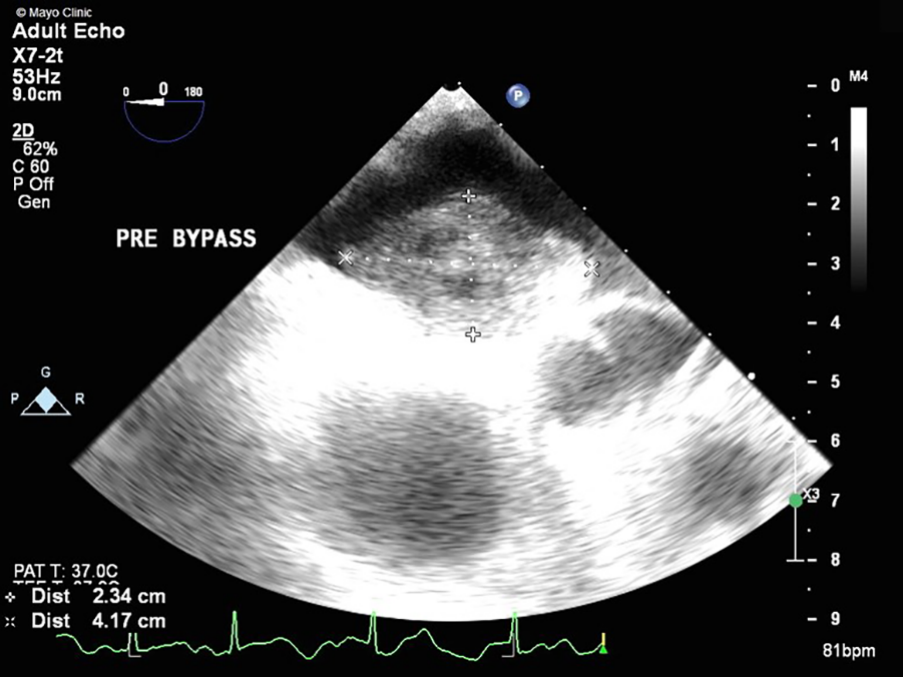
TEE showing a large mass (2.3 cm × 4.2 cm) with attachment to the left atrial side of the atrial septum.

**Figure 2:**
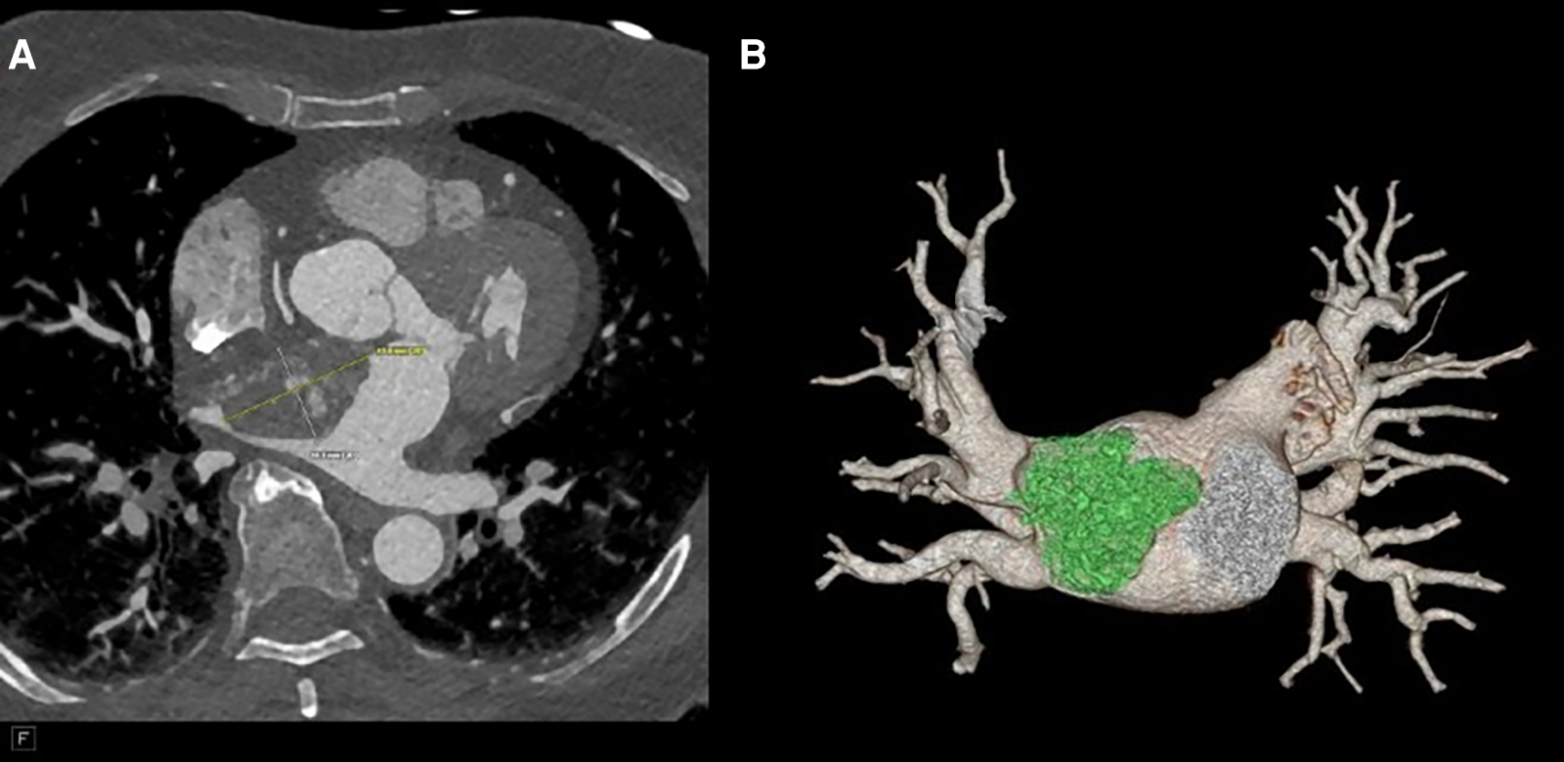
(**A**) CTA revealing a 41 mm × 27 mm × 33 mm broad-based lobulated mass in the left atrium that extends into the mitral annulus. (**B**) CTA reconstruction to show the mass extending to the right upper pulmonary vein (RUPV).

Her physical examination was unremarkable, as exemplified by the fact that her heart sounds were normal. A brain magnetic resonance imaging (MRI) did not show signs of ischemia or prior infarction. An echocardiogram in the ED demonstrated a left atrial mass with a broad base attachment to the atrial septum (Figure 1**)** that extended from the opening of the pulmonary veins to the mitral annulus. Subsequent investigation with coronary computed tomography angiography (CCTA) revealed a 41 mm × 27 mm × 33 mm broad-based lobulated mass in the left atrium that extended onto the mitral annulus (Figure 2). The mass was isointense on T1-weighted images and hyperintense on T2-weighted images and showed restricted diffusion (Supplementary Figure S1). There was heterogeneous enhancement on both early and late postcontrast images. The findings were compatible with a cardiac myxoma (Supplementary Video S1).

### Differential diagnosis

Cardiac myxoma; metastases from unknown primary; undifferentiated high-grade pleomorphic sarcoma; thrombus; leiomyosarcoma; lipoma.

### Management

**Figure 3:**
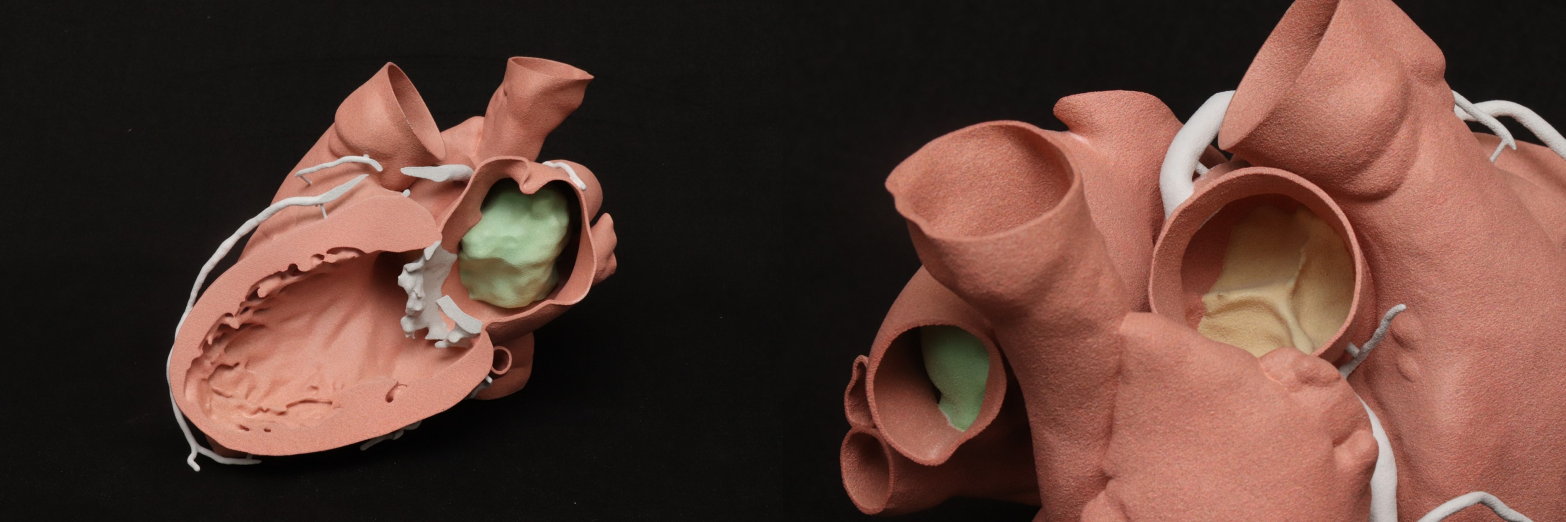
3D-printed models of the myxoma.

Following the reported imaging findings, a careful surgical plan was discussed with the multidisciplinary team for resection of the mass. A CCTA with a three-dimensional (3D) reconstruction of the mass, including a 3D-printed model of the patient's heart depicting the mass and relationship with adjacent structures, was obtained to help with surgical planning (Figure 3). The 3D CT model redemonstrated a polypoid, heterogeneously enhancing left atrial mass attached to the entire left side of the atrial septum and measuring approximately 44 mm × 28 mm × 40 mm. The appearance on the CCTA and the location were consistent with a cardiac myxoma. The mass occluded the right superior pulmonary vein ostium during the atrial systole phase. A large-caliber sinoatrial artery branch arising from the proximal segment appeared to be the primary vascular supply to the mass. A transesophageal echocardiogram (TEE) was obtained, which confirmed that the mass was not adherent to the right superior pulmonary vein (Supplementary Videos S2, S3). Therefore, the patient was deemed to be a surgical candidate for a minimally invasive robotic approach given the location of the mass and the lack of adherence to the wall of the right superior pulmonary vein.

The patient underwent a robot-assisted minimally invasive mass resection. She was placed on cardiopulmonary bypass via the right femoral artery and vein. Access was obtained and the robot was docked to the right chest. After arresting the heart, we elected to access the left atrium via the right inferior pulmonary vein (as per visualization with the 3D-printed model), the point from which the tumor was furthest away. Upon entering the left atrium, a large tumor was identified, and care was taken not to disrupt the tumor. The mass was visualized and was easily separated from the atrial septum (Supplementary Video S4). A partial thickness of the atrial septum was resected along with the mass. The tumor had a gelatinous quality, consistent with that of a myxoma, and was sent to pathology for confirmation (Supplementary Figure S2). In the frozen section, the tumor was reported to be a highly undifferentiated malignant tumor that was adjacent to the line of resection. By the time the frozen section results became available, the atrium had been closed and the cross-clamp was removed, but the patient remained cannulated. With the findings of the pathology, it was decided to reinitiate the cardiopulmonary bypass and arrest the heart again, in order to resect the atrial septum at the foramen ovale, given the potential malignant nature of the tumor. The systemic margins alongside the septum were sent to pathology for evaluation, and the results were negative for malignancy. The atrial septum was closed with a bovine pericardial patch. The patient was extubated in the operating room and had an uncomplicated recovery in the hospital, following which she was discharged 5 days later. The final pathology report confirmed a cardiac myxoma with no malignancy (Supplementary Figure S3). The margins were uninvolved. The tumor cells were shown to be reactive with antibodies directed against PRKAR1A, in keeping with a non-syndromic cardiac myxoma. No complications were encountered by the patient in the postoperative phase.

## Discussion

Cardiac myxomas usually (>90%) occur in an isolated fashion, but rarely, they can also occur in a syndromic context, as part of the Carney complex, an autosomal-dominant condition (3). Cardiac myxomas occurring as part of the Carney complex are termed “syndromic myxomas” and are associated with mutations in *PRKAR1A* (4). In addition to cardiac myxomas, the syndrome is associated with extracardiac myxomas, endocrinopathy, and spotty skin pigmentation (lentiginoses). Cardiac myxomas occurring in the Carney complex are more likely to occur in atypical (non-left atrial) locations, be multiple, and occur earlier in life. Immunohistochemical staining from our patient's myxoma showed positive results for PRKAR1A, but the patient did not have any dermatological manifestations associated with a Carney complex or a history of other tumors.

**Figure 4:**
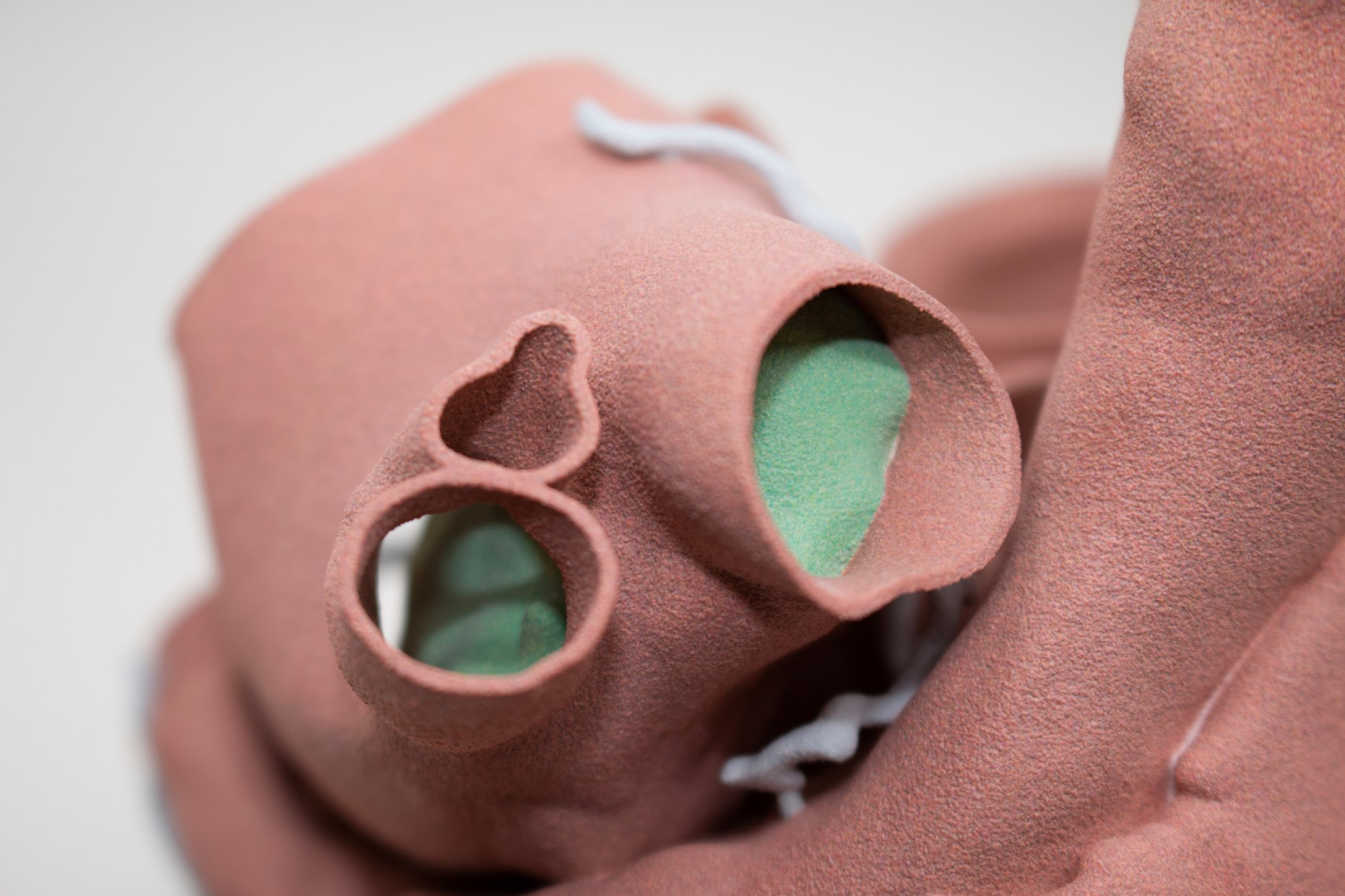
A 3D-printed model showing blood supply to the myxoma in the RUPV.

Myxomas are often initially diagnosed by echocardiography. The classic presentation is a mobile mass on a stalk arising from the atrial septum. In our patient, a TEE was done prior to surgery to assess hemodynamic function. A cavitated sessile mass arising from the atrial septum was seen. The mass did not have a stalk and exhibited contrast within the body of the tumor. The lack of a stalk from which the tumor arose and its cavitated appearance were atypical for a myxoma, warranting further investigation and planning prior to resection. The mass also extended upward into the right pulmonary veins, which is extremely uncommon. Cardiac MRI (CMRI) often complements echocardiography and offers improved tissue characterization, with cardiac myxomas typically demonstrating hypointensity on T1 images, hyperintensity on T2 images, and little to no perfusion or late enhancement (5). T1- and T2-weighted double-recovery sequences aid tissue characterization. Furthermore, cine cardiac imaging holds great importance in evaluating atrial myxomas because of their high mobility and their tendency to prolapse through the atrioventricular valve during diastole (6). Contrast-enhanced sequences are crucial in distinguishing myxomas from thrombus, as myxomas typically exhibit minimal or nil enhancement during first-pass perfusion, yet display a more heterogeneous enhancement pattern on late gadolinium enhancement (LGE) imaging (7). In our case, the patient's cardiac MRI scan did show findings consistent with those of a myxoma, but it revealed more enhancement than is typical. Cardiac MRI has shown remarkable accuracy in identifying cardiac masses and effectively differentiating between benign and malignant tumors. However, relying solely on MRI has been associated with occasional instances of inaccurate diagnosis. Therefore, adopting a multimodal imaging approach comprising echocardiography, MRI, CT, and possibly positron emission tomography (PET) imaging offers an optimal approach for a comprehensive evaluation and stratification of cardiac masses (8). Given the atypical characteristics and location of our patient's left atrial mass, a CT angiogram with a 3D reconstruction of the heart depicting the mass was requested to guide the diagnosis and surgical planning. The mass was heterogeneously enhancing and polypoid in shape. The mass did not infiltrate or invade surrounding structures. This observation holds considerable significance as it reduces the likelihood of the tumor being a malignant one (9). However, it did extend to the ostium of the right superior pulmonary vein, occluding it during atrial systole. It is hard to ascertain whether the mass was the source of our patient's symptoms, but these findings could potentially explain the patient's orthostatic syncope and dizziness. A case report in the literature describes a patient presenting with syncope and dyspnea, who was found to have a left atrial myxoma extending into and occluding the left pulmonary veins and causing pulmonary infarction (10). However, in our patient, the mass did not extend or occlude the mitral valve, which made it feasible to adopt a robotic approach. Furthermore, the mass received blood supply from the small sinoatrial node branch of the right coronary artery. This was visualized precisely and with high spatial resolution on the 3D-printed model, which also showed the extent of expansion of the mass into the right upper pulmonary veins (Figure 4). The printed model also provided for visualization of the best approach to enter the left atrium without disruption of the mass. A possible implication of this occurrence is the formation of a fistula between the sinoatrial nodal artery and the right atrium after surgical resection of the atrial myxoma. For this reason, special attention should be paid to ligating neovascularized branches feeding myxomas during the surgical procedure (11).

## Conclusion

This study described a peculiar and unique presentation of a left atrial myxoma extending into the right upper pulmonary veins. Multimodal imaging, including 3D reconstruction and printing of the heart and mass, guided the diagnostic approach and successful resection of the mass by robotic intervention.

## Data Availability

The original contributions presented in the study are included in the article/**Supplementary Material**, and further inquiries can be directed to the corresponding author.

## Supplementary material

Supplementary Figure S1 Cardiac MRI images depicting (**A**) T1-weighted and (**B**) T2-weighted sequences.

Supplementary Figure S2 Gross photograph of a resected cardiac tumor. (**A**) The tumor has a lobulated morphology and (**B**) in the cut section has a gelatinous quality.

Supplementary Figure S3 Photomicrograph of a resected cardiac tumor. (**A**) Hematoxylin and eosin staining discloses bland spindle-shaped cells, occurring singly and in small clusters, proliferating in a myxoid background, consistent with a cardiac myxoma. (**B**) The neoplastic cells are reactive with antibodies directed against PRKAR1A, suggesting a non-syndromic tumor.

Supplementary Video S1 A four-chamber view shows the entire left atrial septum being engulfed by the large mass and extending up and into the right upper pulmonary vein.

Supplementary Video S2 A TEE of a myxoma four-chamber. (**A**) A demonstration of the entire left atrial septum being engulfed by the tumor mass. (**B**) A zoom view showing flow within the mass by a color Doppler.

Supplementary Video S3 A TEE at 70°–88° showing the myxoma filling the right superior pulmonary vein (**A**); color (**B**) freely moving and color flow around the mass.

Supplementary Video S4 A robot-assisted minimally invasive resection of an atypical left atrial myxoma.
