# Supplementary figures and images for: Case Report: A myxoma with a far reach
Source: Front Cardiovasc Med. 2024 Jan 24;11:1340406. doi: 10.3389/fcvm.2024.1340406 (PMC10847281; doi:10.3389/fcvm.2024.1340406)

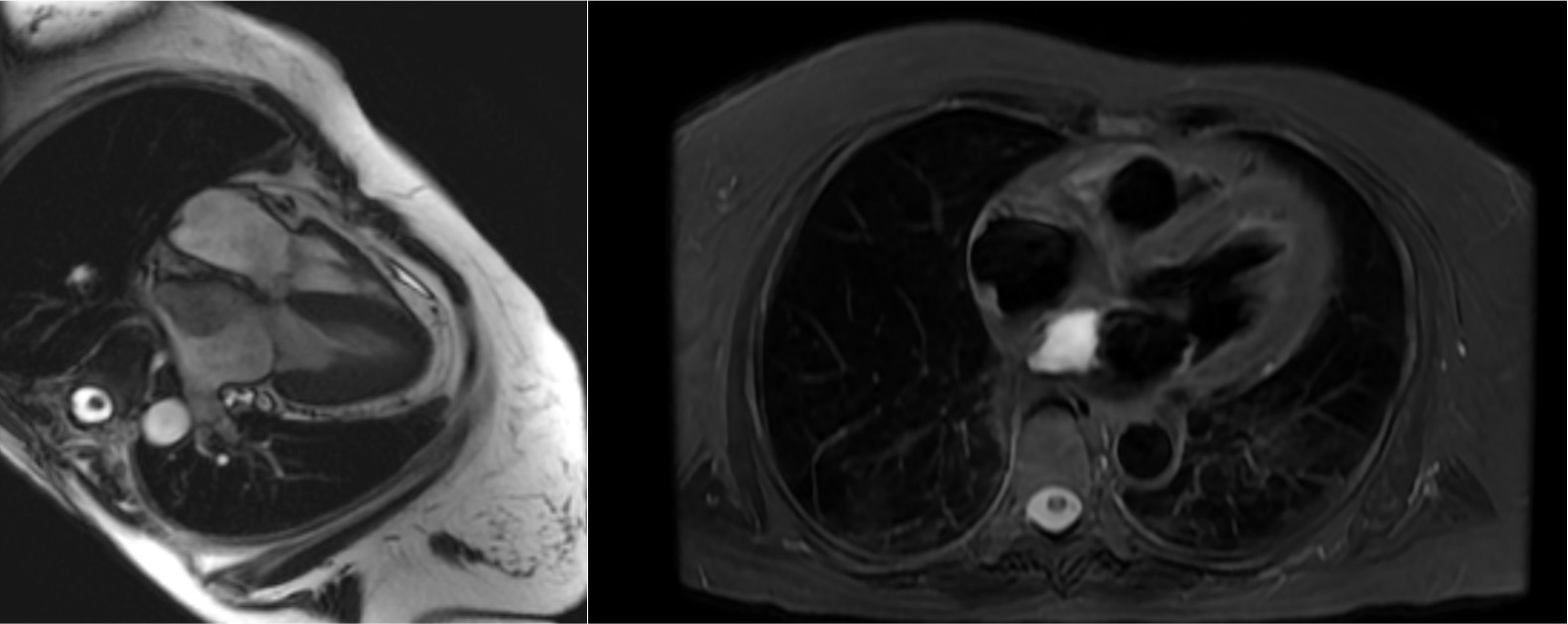

Supplement: Supplementary Figure S1 — Cardiac MRI images depicting (A) T1-weighted and (B) T2-weighted sequences. [file Image1.jpeg]

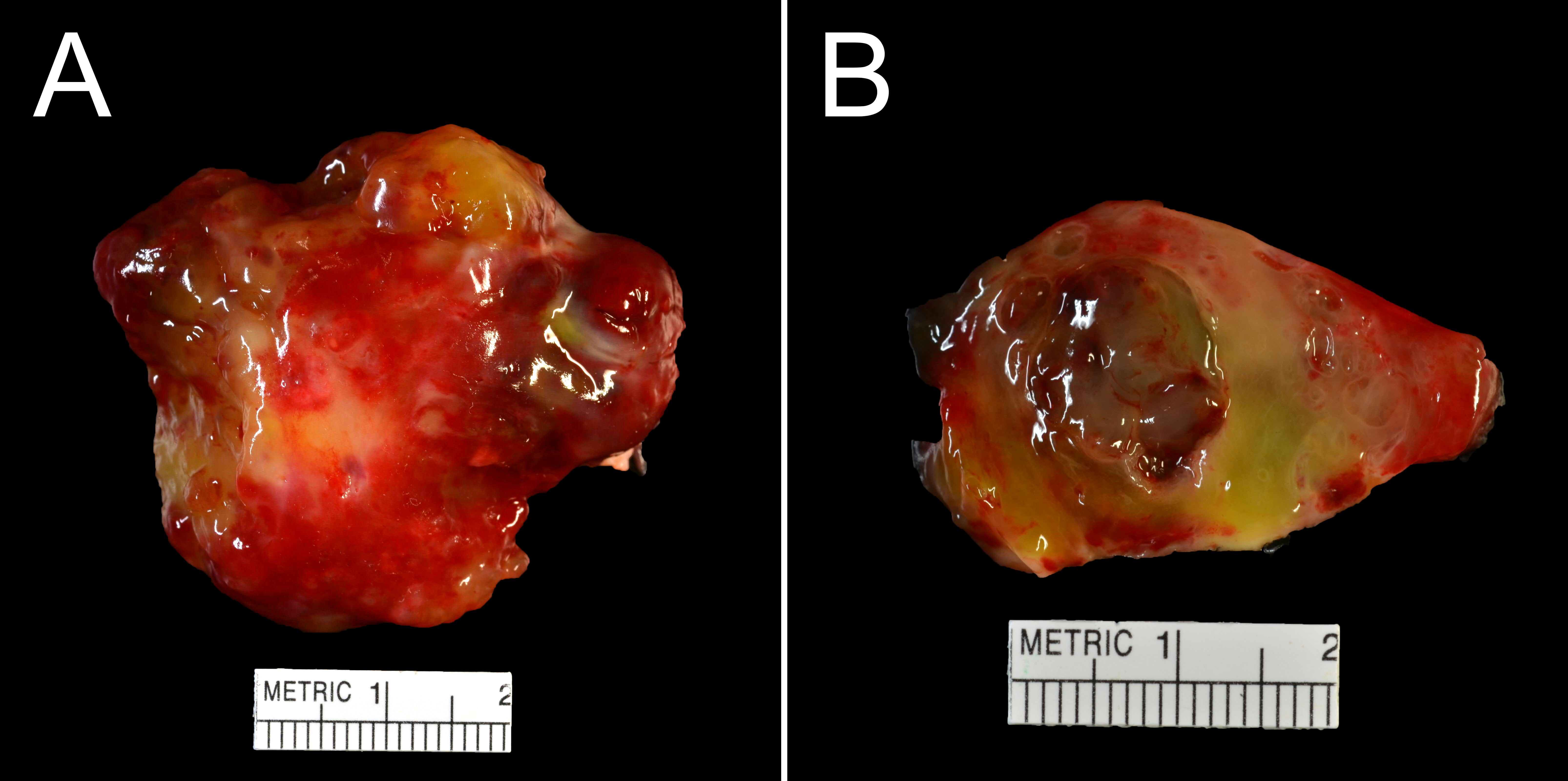

Supplement: Supplementary Figure S2 — Gross photograph of a resected cardiac tumor. (A) The tumor has a lobulated morphology and (B) in the cut section has a gelatinous quality. [file Image2.jpeg]

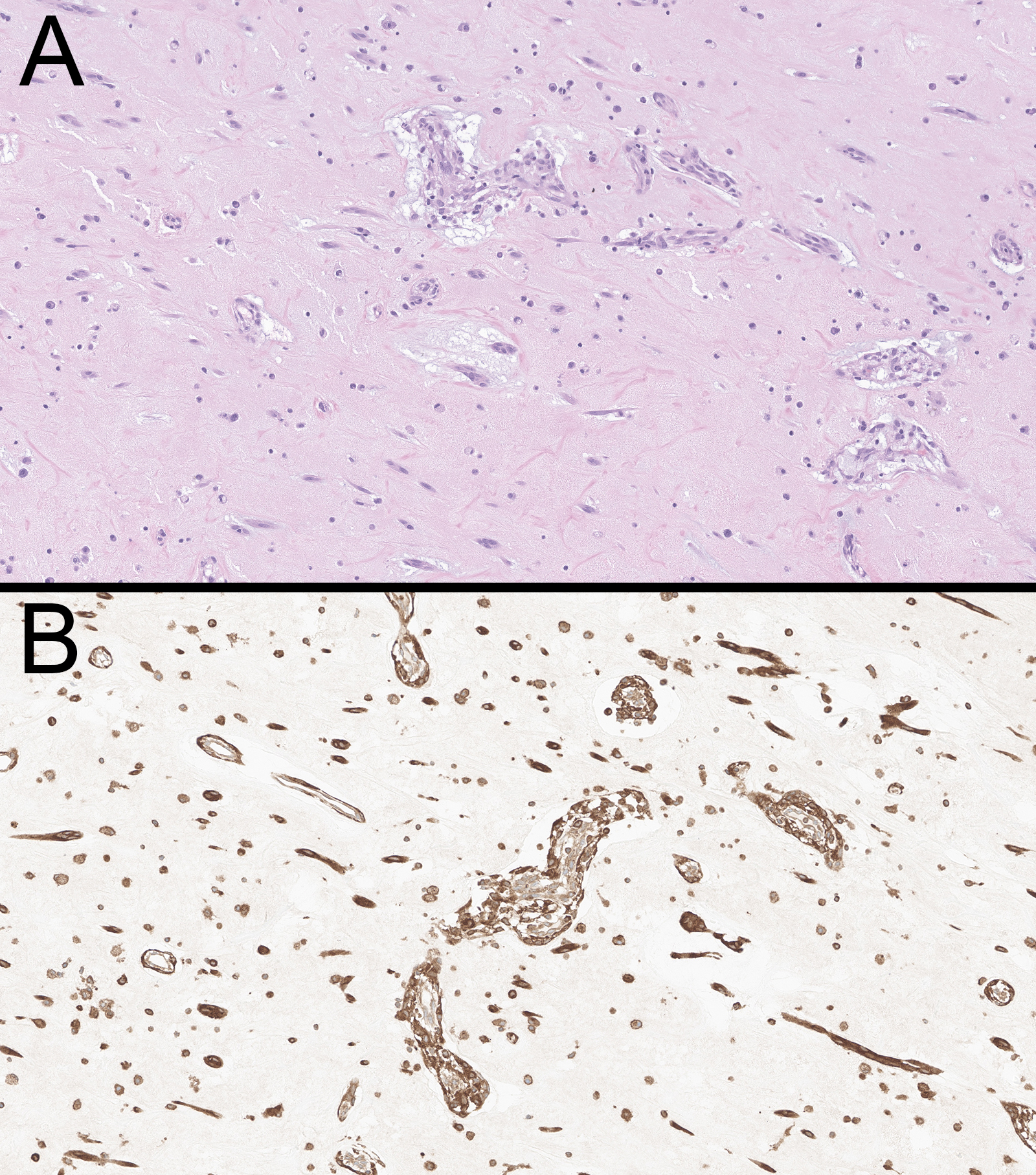

Supplement: Supplementary Figure S3 — Photomicrograph of a resected cardiac tumor. (A) Hematoxylin and eosin staining discloses bland spindle-shaped cells, occurring singly and in small clusters, proliferating in a myxoid background, consistent with a cardiac myxoma. (B) The neoplastic cells are reactive with antibodies directed against PRKAR1A, suggesting a non-syndromic tumor. [file Image3.jpeg]
